# Effect of Polyherbal Mixtures on the Treatment of Diabetes

**DOI:** 10.1155/2013/934797

**Published:** 2013-04-16

**Authors:** Aparajeya Panda, Somanatha Jena, Pramod Kumar Sahu, Sanghamitra Nayak, Payodhar Padhi

**Affiliations:** ^1^Hi-Tech Research and Development Centre, Konark Institute of Science and Technology, Bhubaneswar, Odisha 752050, India; ^2^P.G. Department of Botany, Utkal University, Bhubaneswar, Odisha 751004, India; ^3^Department of Biotechnology, Rama Devi Women's (Auto) College, Bhubaneswar, Odisha 751022, India; ^4^Centre for Biotechnology, SOA University, Bhubaneswar, Odisha 751003, India

## Abstract

The study focuses on polyherbal antidiabetic formulations of different plants used in the treatment of diabetes mixed in different concentrations. In the present study eleven medicinal plants with proven antidiabetic and related beneficial effects were selected for the preparation of five mixtures. The efficacy of prepared mixtures has been tested on streptozotocin- (STZ-) induced diabetic rats and compared with a commercially available drug glibenclamide. The mixtures at the dose levels of 400 mg/kg b.w. produced a significant decrease in blood glucose level by 69.6%, 70.97%, 64.45%, 71.82%, and 64.44% after 21 days of treatment. The elevated level of SGPT, SGOT, and ALP in the diabetic controlled group reflected the significant alteration of liver function by STZ induction and was found to be equipotent to glibenclamide in restoration of the elevated enzyme levels to normal. The elevated lipid levels (triglyceride and total cholesterol) were restored to near normal by these mixtures for all the estimated parameters. The results of the mixtures on treated group were found to restore the glycemic level to the near normal level thereby indicating antihyperglycemic activity of the formulated mixtures.

## 1. Introduction

At present there is an extensive growth in the field of herbal mixtures, and these mixtures are gaining popularity both in developing and developed countries because of their natural origin and less side effects. Many traditional medicines are derived from medicinal plants and minerals which are used for the treatment of different chronic diseases like diabetes, asthma, and so forth [1]. Major hindrance in amalgamation of herbal medicine in modern medical practices is lack of scientific and clinical data proving their efficacy and safety. There is a need for conducting clinical research in herbal mixtures, developing simple bioassays for biological standardization, pharmacological and toxicological evaluation, and developing various animal models for toxicity and safety evaluation. It is also important to establish the active components of these herbal extracts [2].

Diabetes is a metabolic disorder characterized by increased fasting and postprandial blood sugar levels. The prevalence of diabetes is likely to be increased by 35% [2]. It may be projected from 15 million in 1995 to 57 million in 2025 [3]. In the present study eleven plants (cited below) have been selected for preparation of five mixtures. The *Ferula assa-foetida *is found to reduce the body weight of alloxan-induced albino rats [4]. The *Annona squamosa* extract brings blood glucose levels, serum insulin levels, serum lipid profiles, and body weight to normal level in streptozotocin-nicotinamide-induced diabetic rats [5]. Fresh juice of *Zingiber officinale* produced a time dependent decrease in blood glucose level significantly compared to both glibenclamide and metformin in STZ-induced diabetic rats [6]. The leave extracts of *Gymnema sylvestre* show significant reduction in blood glucose, glycosylated haemoglobin, and glycosylated plasma proteins in diabetic patients [7]. Aqueous extract of seeds of *Tamarindus indica *Linn. was found to have potent antidiabetogenic activity that reduces blood sugar level in streptozotocin- (STZ-) induced diabetic male rat [8]. The aqueous extract of *Azadirachta indica* lowers the blood glucose and improves the body weight gain in streptozotocin-induced diabetic rats [9]. The alcoholic seed extract of *Trigonella foenum-graecum* lowers the blood glucose level in alloxan-induced diabetic rats [10]. The ethanolic and aqueous extracts of roots of *Moringa oleifera* lower the blood glucose level in streptozotocin- (STZ-) induced diabetic rats [11]. The aqueous extract of *Aegle marmelos* seeds shows antidiabetic and hypolipidemic effects in streptozotocin-induced diabetic rats [12]. The methanolic leave extracts of *Cajanus cajan* reduce fasting blood sugar level in alloxan-induced diabetic rats [13]. Administration of the aqueous extracts of *Cinnamomum tamala* (CTLEt) leaves decreases the levels of fasting blood glucose and urine sugar, with a concomitant increase in body weight in streptozotocin-induced diabetic rats [14].

Keeping on view of the above effectiveness of the herbals for diabetics, it is proposed to formulate polyherbal mixtures that is combination of the above herbal parts which were studied and the efficacy of mixtures compared with marketed drug glibenclamide.

## 2. Material and Methods

### 2.1. Herbal Materials

Eleven herbal parts having antidiabetic activity were selected from the literature [4–14]. Five different mixtures were formulated by using different ratios of eleven different types of plant parts at random. The compositions of the mixtures are given in Table 1.

### 2.2. Preparation of Polyherbal Extracts

The fresh leaves/seeds were plucked and separated from the twigs. These were washed clearly, shade-dried, and then ground by a mechanical grinder. The coarse powder was extracted with distilled water using soxhlet at boiling temperature (60°C–80°C) up to 10 h. A dark brown coloured extract was obtained. This dark brown extract was cooled and filtered to remove the residue. The extract was concentrated on rotary evaporator under reduced pressure and then dried to get a powder. The dried powder was diluted with saline in required proportion for the study. 

### 2.3. Acute Toxicity Studies

The lethal dose (LD_50_) of the plant extract was assessed by using albino mice of either sex weighing 20–25 g to determine the dose. The animals were fasted overnight prior to the experimental procedures. Different doses of extract were administered by the intraperitoneal route. The LD_50_ was calculated according to Miller and Tainter [15]. 1/10th of lethal dose was taken as a screening dose [16].

### 2.4. Experimental Animals

The Wistar albino rats (200–220 g) and Swiss albino mice of both sexes (20–25 g) were purchased from the animal house of Orissa University of Agriculture and Technology, Bhubaneswar, India. They had free access to standard rat pellets and water *ad libitum* and were maintained under standard condition at a temperature of 25 ± 2°C, with a 12/12 light/dark cycle and 35–60% humidity.

### 2.5. Induction of Type II Diabetes

Diabetes was induced in experimental rats by single intraperitoneal injection of streptozotocin (80–120 mg/kg body weight/day) dissolved in 0.1 M citrate buffer (pH 4.5). Diabetes was confirmed in animals having blood glucose level more than 230 mg/kg body weight after 48 hours of STZ administration, and blood samples were analyzed by the Accu-Chek active blood glucose meter for estimation of blood glucose levels. Animals having fasting blood glucose levels higher than 230 mg/dL were considered for experiments.

### 2.6. Experimental Design

Animals were divided into eight groups of six rats each. The LD_50_ value of mixtures was 600 mg per kg body weight of mice. The mixtures were administered orally 400 mg per kg body weight once a day for 21 days (Table 2).

### 2.7. Statistical Analysis

The results are expressed as mean ± SD. Statistical evaluation of the data was done by one-way ANOVA followed by Dunnett's *t*-test. *P* values less than 0.05 were considered to be significant.

### 2.8. Estimation of Biochemical Parameters

The blood samples were collected on the 22nd day from the retroorbital plexus of the rats, serum was separated, and the biochemical estimations of serum glutamic pyruvic transaminase (SGPT), serum glutamic oxaloacetic transaminase (SGOT) [17], alkaline phosphatase (ALP) [18], total protein [19], total cholesterol, and triglyceride [20]. 

## 3. Results and Discussions

**Figure 1:**
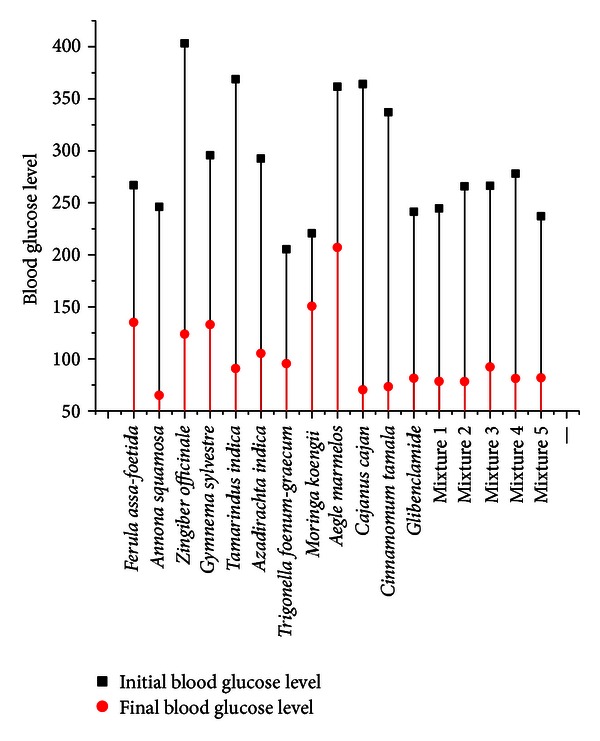
Name of the herbals and polyherbal mixtures.

The blood glucose level observations have been recorded in Table 3, and the effect of the mixtures on the body weight of rat has been shown in Table 4. The effect of multiherbal extracts on some biochemical parameters like SGPT, SGOT, ALP, total protein, triglyceride, and total cholesterol in control and STZ-induced diabetes rats has been reported in Table 5. A comparison graph between individual herbals and multiherbal extracts has been shown in Figure 1. 

Here we evaluated the hypoglycemic activity of the aqueous extracts of prepared in-house mixtures and the marketed drug in streptozotocin-induced diabetic rat. From Figure 1 it can be observed that the multiherbal extracts have maintained the blood glucose level below 100, which is a better result in comparison to individual herbals. As shown in Table 3, the formulated mixtures significantly reduced the blood glucose level in STZ-induced diabetic rat. The hypoglycemic activity of mixture 1, 2, 3, 4, and 5 and glibenclamide showed 69.6%, 70.97%, 64.45%, 71.82%, 64.44%, and 64.91% antidiabetic activity, respectively. All the mixtures have shown potential in their role to reduce the blood glucose level. Mixture 4 showed higher activity as compared to the other mixtures (Table 3). This may be because of the fact that some of the herbal counterparts in mixture 4 (Table 1) were not present in the others which may possess a higher antidiabetic activity. The formulated mixtures also prevent the loss of body weight in comparison to the standard drug glibenclamide (Table 4).

Diabetes is associated with alternation of plasma lipid and lipoprotein and is consequently linked to increased risk of coronary heart disease [21]. Insulin deficiency and increased blood glucose level lead to hyperglycemia and hypercholesterolemia, as was found in the diabetic control group in the present study. This is may be due to uninhibited actions of lipolytic hormones on the fat depots and increased mobilization of free fatty acids from the fat depot. This excess fatty acid is converted into phospholipids and cholesterol in liver. The elevated lipid levels were restored to near normal in the multiherbal extracts treated groups.

The elevated levels of SGPT,  SGOT, ALP, total protein, total cholesterol, and triglyceride in the diabetic control group reflected the significant alteration of liver function by STZ induction (Table 5). The extract was found to be equipotent to glibenclamide in restoration of the elevated enzyme levels to normal, implying the normal functioning of liver. Several authors have reported that flavonoids, tannins, alkaloids, and terpenes are known to be bioactive antidiabetic principles [22–24]. The antihyperglycemic effect of these mixtures may be due in part to their flavonoids, alkaloids, tannins, and terpenes.

## 4. Conclusions

The formulated mixtures on treated group were found to restore the glycemic level to the near normal level thereby indicating antihyperglycemic activity of the formulated mixtures. These formulations also restore the SGPT, SGOT, and ALP levels which indicate that they reduce the other complicacies of diabetes. A deeper insight into these herbs may lead to development of more potent antidiabetic formulations.

## Figures and Tables

**Table 1:** Composition of mixtures.

Sl. no.	Herbals	Quantity of herbals in different mixtures (in gm)				
		Mixture 1	Mixture 2	Mixture 3	Mixture 4	Mixture 5
1	*Ferula assa-foetida *	10	5	10	5	5
2	*Annona squamosa *	80	55	30	45	35
3	*Zingiber officinale *	45	80	30	55	50
4	*Gymnema sylvestre *	40	45	30	35	50
5	*Tamarindus indica *	30	45	80	40	55
6	*Azadirachta indica *	35	50	65	80	30
7	*Trigonella foenum-graecum *	70	40	80	55	30
8	* Moringa oleifera *	45	70	55	40	80
9	*Aegle marmelos *	50	35	40	45	55
10	*Cajanus cajan *	40	30	50	60	35
11	*Cinnamomum tamala *	55	45	30	40	75

**Table 2:** Administration of mixtures in diabetic rat.

Sl. no.	Groups	Type of mixture administered in rat
1	Group I	2 mL sterilized distilled water per kg body weight
2	Group II	2 mL sterilized distilled water per kg body weight
3	Group III	Glibenclamide (10 mg per kg body weight)
4	Group IV	Mixture 1
5	Group V	Mixture 2
6	Group VI	Mixture 3
7	Group VII	Mixture 4
8	Group VIII	Mixture 5

**Table 3:** Antihyperglycemic activity of mixtures on STZ-induced diabetic rat.

Group (*n* = 6)	Dose (mg/kg)	Blood glucose level (mg/dL)						
		0 h	2 h	4 h	8 h	12 h	21 days	*F* value
Normal control	2 mL/kg	92.46 ± 3.59	90.66 ± 2.57	92.75 ± 4.53	89.66 ± 2.72	91.67 ± 4.66	90.68 ± 3.22	8.05
Diabetic control	2 mL/kg	234.46 ± 3.59	240.66 ± 2.57	237.54 ± 2.53	241.54 ± 2.66	259.21 ± 3.44	255.3 ± 2.11	0.55
Glibenclamide	10	241.33 ± 4.26**	201.83 ± 3.65**	162.56 ± 2.33**	125.33 ± 2.32**	84.66 ± 3.11**	81.33 ± 2.12**	130.58
Mixture 1	400	244.60 ± 2.6**	188.8 ± 3.33**	168.33 ± 2.33**	111.83 ± 2.33**	74.33 ± 3.33**	78.43 ± 2.11**	127.32
Mixture 2	400	265.83 ± 2.33**	196.8 ± 3.33**	155.33 ± 2.33**	107.33 ± 2.33**	77.16 ± 2.67**	78.11 ± 2.11**	121.55
Mixture 3	400	266.32 ± 2.33*	222.6 ± 2.67*	169.33 ± 2.33*	127.16 ± 2.67*	94.66 ± 2.67*	92.12 ± 3.12*	119.32
Mixture 4	400	278.00 ± 2.55**	151.00 ± 2.33**	135.83 ± 2.33**	128.83 ± 2.33**	78.33 ± 3.33**	81.21 ± 4.11**	100.43
Mixture 5	400	237.16 ± 2.67*	162.6 ± 3.67*	138.66 ± 2.67*	108.66 ± 2.67*	84.33 ± 3.33*	81.76 ± 1.21*	118.43

Results expressed as mean ± SD (*n* = 6). Treatment was done for 21 days. The data were statistically analysed by one-way ANOVA, followed by Dunnett's *t*-test. *P < 0.01, **P < 0.05. *P* values less than 0.01 were considered more significant.

**Table 4:** Effect of mixtures on body weight of STZ-induced diabetic rat.

Groups	Initial weight (gm)	Final weight (gm)	Change in body weight	Change in body weight (%)
Normal control	210.00 ± 8.91	255.17 ± 678	45.17 ± 6.78	+28.23 ± 3.85
Diabetic control	231.17 ± 8.78	212.00 ± 8.42	−19.17 ± 5.21	−11.15 ± 2.68
Diabetic + glibenclamide	218.00 ± 7.89**	256.50 ± 8.16**	38.50 ± 5.98**	+22.65 ± 1.08**
Diabetic + mixture 1	200.84 ± 7.75*	222.49 ± 6.78*	21.65 ± 8.9*	+12.45 ± 2.96*
Diabetic + mixture 2	220.13 ± 8.75*	243.91 ± 5.67*	23.78 ± 5.81*	+12.50 ± 3.86*
Diabetic + mixture 3	200.17 ± 7.45**	226.35 ± 6.02**	26.18 ± 6.21**	+14.37 ± 1.96**
Diabetic + mixture 4	205.83 ± 7.33*	221.48 ± 7.66*	15.65 ± 8.88*	+9.10 ± 1.23*
Diabetic + mixture 5	208.87 ± 7.75*	229.52 ± 7.88*	20.65 ± 5.83*	+11.54 ± 1.05*

Results expressed as mean ± SD (*n* = 6). Treatment was done for 21 days. The data were statistically analysed by one-way ANOVA, followed by Dunnett's *t*-test. *P < 0.01, **P < 0.05. *P* values less than 0.01 were considered more significant.

**Table 5:** Effect of multiherbal extracts on biochemical parameters in control and STZ-induced diabetic rats.

Groups	SGPT (IU/dL)	SGOT (IU/dL)	ALP (IU/dL)	Total protein (mg/dL)	Triglyceride (mg/dL)	Total cholesterol (mg/dL)
Normal control (2 mL/kg)	122.22 ± 2.54	130.51 ± 3.77	55.99 ± 3.99	8.30 ± 1.87	84.55 ± 2.71	138.6 ± 3.11
Diabetic control (2 mL/kg)	214.33 ± 4.40	250 ± 6.02	90.5 ± 4.53	6.16 ± 1.93	214.15 ± 5.10	253.83 ± 6.16
Glibenclamide (10 mg/kg)	110.66 ± 3.13**	140.16 ± 4.29**	46.83 ± 4.29**	8.16 ± 1.46**	109.66 ± 3.76**	124 ± 5.92**
Mixture 1 (400 mg/kg)	130.00 ± 4.46**	160.16 ± 3.64**	51.33 ± 5.45**	5.83 ± 2.21*	130.83 ± 4.82**	146.16 ± 5.33**
Mixture 2 (400 mg/kg)	121.16 ± 4.15**	149.33 ± 5.74**	49.83 ± 5.74**	7.66 ± 2.15*	121.33 ± 4.31**	138.5 ± 4.49**
Mixture 3 (400 mg/kg)	117.00 ± 3.39**	142.33 ± 5.30**	47.5 ± 4.66**	8 ± 2.27**	112.5 ± 4.79**	131.16 ± 5.69**
Mixture 4 (400 mg/kg)	118.5 ± 3.44**	138.98 ± 3.77**	40.62 ± 3.65**	5.66 ± 2.54*	118.34 ± 3.56**	122.44 ± 4.23**
Mixture 5 (400 mg/kg)	119.33 ± 3.56**	145 ± 3.87**	48.4 ± 5.23**	6.67 ± 4.33*	120.5 ± 4.89**	135.5 ± 4.33**

Results expressed as mean ± SD (n = 6). Treatment was done for 21 days. The data were statistically analysed by one-way ANOVA, followed by Dunnett's t-test. *P < 0.01, **P < 0.05. P values less than 0.01 were considered more significant.
